# Multimorbidity patterns and cognitive trajectories in older adults: functional, behavioral, and environmental pathways—a longitudinal analysis of CHARLS

**DOI:** 10.21203/rs.3.rs-10231629/v1

**Published:** 2026-09-18

**Authors:** Yanhui Jiao, Jiaxin Li, Xiaohan Sun, Qing Li, Xiaohui Zhai

**Affiliations:** Chinese Academy of Medical Sciences & Peking Union Medical College; Cangzhou Central Hospital Yanshan Branch; Jitang College of North China University of Science and Technology; Chinese Academy of Medical Sciences & Peking Union Medical College; National Health Commission of the People’s Republic of China

**Keywords:** Multimorbidity, Cognitive trajectories, Latent class analysis, Latent growth curve model, CHARLS, Aging

## Abstract

Multimorbidity may affect cognitive aging differently according to disease patterns rather than disease counts alone. This study analyzed 2011–2020 data from the China Health and Retirement Longitudinal Study, including 5979 Chinese adults aged 45 years or older. Fifteen chronic conditions were used to identify multimorbidity patterns through latent class analysis. Latent growth curve models examined associations with baseline cognition and cognitive decline, while mediation and moderation analyses assessed activities of daily living, social and intellectual engagement, living environment quality, and adverse childhood experiences. Five patterns were identified: minimal morbidity, sensory-psychiatric-musculoskeletal multimorbidity, cardiometabolic multimorbidity, extensive multisystem multimorbidity, and respiratory-psychiatric-sensory multimorbidity. In fully adjusted models, sensory-psychiatric-musculoskeletal multimorbidity (β = −0.757; 95% CI, − 0.92 to − 0.59) and extensive multisystem multimorbidity (β = −0.469; 95% CI, − 0.73 to − 0.21) were associated with lower baseline cognition. Both showed slower subsequent decline, suggesting a floor effect. Time-varying models showed faster cognitive decline in the cardiometabolic group (β = −0.028; 95% CI, − 0.052 to − 0.005; P = 0.019). Activities of daily living mediated associations across nonreference patterns, whereas social and intellectual engagement mediated only the sensory-psychiatric-musculoskeletal pattern. Poor living environment quality was associated with lower baseline cognition (β = −0.532; 95% CI, − 0.849 to − 0.214; P = 0.002). These findings support phenotype-aware cognitive risk screening and interventions targeting function, engagement, and living environments.

## Background

By 2050, approximately one in six people worldwide will be over 65 years of age [1]. This demographic shift is already pushing age-related cognitive decline to the forefront of public health, with dementia now affecting more than 55 million individuals and Alzheimer’s disease accounting for the bulk of the burden—approximately 10 million new cases every year[2]. Multimorbidity has become the norm in later life, and it is closely associated with poorer cognitive performance, faster decline, and elevated dementia risk[3]. However, simply counting diseases or relying on composite comorbidity indices often masks the clinically important heterogeneity that distinguishes one cluster of chronic conditions from another[4, 5]. Diseases do not accumulate at random; they coalesce into recognizable patterns, each carrying its own biological signature and functional footprint[6]. Capturing these latent structures should, in theory, flag cognitive risk more effectively than crude tallies, allowing for a far more nuanced reading of how specific disease combinations shape cognitive outcomes.

Nevertheless, the temporal relationship between multimorbidity patterns and cognitive aging remains unclear. Most studies have looked at cognition at a single point or over short follow-ups, usually failing to separate baseline cognitive levels from the pace of subsequent change[7]. However, different profiles may have distinct temporal signatures—some might depress initial performance, while others could accelerate the decline[8]. Disentangling these possibilities calls for longitudinal designs that explicitly separate starting cognition from its trajectory. Another gap is equally important: most investigations have treated multimorbidity as a fixed baseline exposure, ignoring the added risks that emerge as disease configurations shift during follow-up. By modeling time-varying comorbidity patterns within linear mixed-effects frameworks, one can capture the evolving interplay between dynamic disease transitions and both the concurrent cognitive level and the rate of change.

Moreover, the mechanisms linking multimorbidity patterns to cognitive aging are still largely unclear. The disablement process framework posits that accumulating chronic diseases erode physical reserves, compromise activities of daily living (ADLs), and ultimately reverberate through cognitive health[9]. Functional limitations, sensory impairment, and emotional distress can also constrict social interaction and curtail participation in cognitively stimulating pursuits, thereby depleting the very stimulation and support that help sustain cognition[10]. Until recently, however, physical function and social participation have usually been studied in silos; few investigations have considered ADL function and engagement in social and intellectual activities as parallel explanatory pathways running from multimorbidity profiles to cognitive trajectories[11]. Beyond the individual, the surrounding living environment adds another layer. Housing quality, clean fuel access, tap water availability, and ambient air pollution shape physical function, mobility, social engagement, and ultimately cognitive well-being[12, 13]. In addition, early life experiences, in particular, childhood adversity, may scar cognitive reserve and thereby modulate how strongly multimorbidity forecasts decline[14]. Longitudinal evidence that clarifies these moderating roles remains scarce.

We aim to address these research gaps by combining five waves of the China Health and Retirement Longitudinal Study (2011–2020) with city-level air-pollution data. We wanted to determine what happens when disease patterns, cognitive trajectories, functional and behavioral pathways, and environmental context are examined together. Specifically, we used latent class analysis (LCA) to identify multimorbidity profiles and latent growth curve models (LGCMs) to chart changes in cognition from 2011 to 2020. We then tested whether ADL function and social and intellectual engagement act as parallel mediators and whether living environment quality (LEQ) and adverse childhood experiences (ACEs) modify the picture. We also treated wave-specific multimorbidity patterns as time-varying exposures, allowing us to gauge how dynamic disease transitions are related to cognitive level and change as they occur. By weaving these pieces together, we hope to give clinicians and policymakers something more concrete than another disease count tally.

## Methods

### Research design and content

We drew on data from the 2011, 2013, 2015, 2018, and 2020 waves of the China Health and Retirement Longitudinal Study (CHARLS), a nationally representative prospective cohort of Chinese adults aged 45 years and older spanning 28 provinces[15]. CHARLS relies on a multistage, stratified, probability-proportional-to-size sampling strategy, in which detailed information on health, economic, social, and cognitive domains is gathered. Ethics approval was granted by the Review Committee of Peking University for all waves, and every participant provided written informed consent.

**Figure 1:**
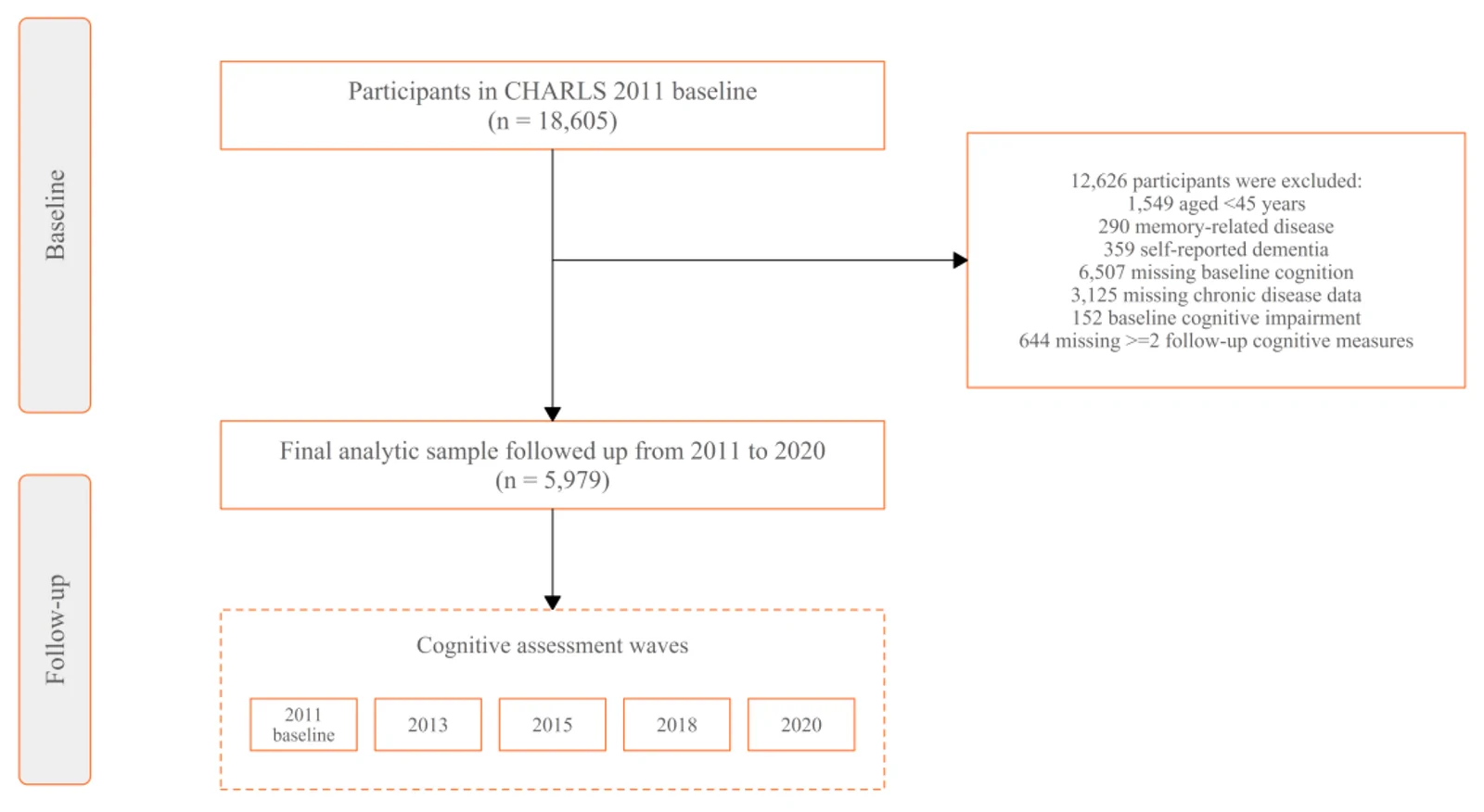
Flow diagram for participants selection in the study. The flowchart details the exclusion criteria applied to the initial China Health and Retirement Longitudinal Study (CHARLS) 2011 baseline cohort to reach the final analytical sample (n = 5,979). The bottom panel displays the five cognitive assessment waves conducted during the 2011–2020 follow-up period.

We excluded individuals younger than 45 years at baseline; individuals who reported a physician diagnosis of dementia, Parkinson’s disease, or another memory-related disorder; anyone missing baseline cognitive or chronicdisease data; participants with baseline cognitive impairment (total score <5); and individuals lacking cognitive assessments in two or more follow-up waves. After applying these criteria, 5,979 participants were included in our analyses (Fig. 1).

Air pollution data were obtained from the China High Air Pollutants (CHAP) database, which supplies annual ambient PM2.5 concentrations at a 1 km resolution for mainland China starting in 2000[16]. Because CHARLS withholds residential addresses for privacy protection, we used city-level annual mean PM2.5 as a proxy for individual exposure, aligning each survey year with the corresponding or preceding annual average (for instance, the 2010 mean was matched to the 2011 wave to allow for lag effects). Reporting follows the STROBE guidelines[17].

### Variable description

#### Exposure: multimorbidity patterns

Our exposure included fifteen chronic conditions: hypertension, diabetes, dyslipidemia, heart disease, stroke, chronic lung disease, asthma, arthritis, digestive disease, liver disease, kidney disease, cancer, psychiatric and depressive disorders, hearing impairment, and visual impairment. Hearing impairment was defined as self-reported hearing loss, poor hearing, or current hearing aid use; visual impairment was defined as self-reported blindness or poor vision[18]. We ascertained all other conditions through self-reported physician diagnosis or current medication use. Each condition was coded as binary (1 = present, 0 = absent). For descriptive purposes, multimorbidity meant having two or more of the aforementioned conditions simultaneously. In the LCA, we retained every baseline participant—including those free of any chronic condition—so that the resulting low-burden class could serve as a natural reference. The 2011 LCA solution anchored the primary analysis, with each participant assigned to the class carrying the highest posterior probability. Because psychiatric and depressive disorders were already embedded in the LCA indicators, we deliberately omitted depressive symptoms from subsequent models to avoid overadjusting for an exposure component.

#### Outcome: cognitive function

Cognitive function was assessed at every wave through interviewer-administered tests: mental status and attention via the 10-item Telephone Interview for Cognitive Status (TICS-10), episodic memory via immediate and delayed word recall, and visuospatial ability via a figure-drawing task. Global scores ranged from 0 to 21, with higher values indicating better cognition. Because word-recall procedures in 2018 and 2020 diverged from earlier waves, we first harmonized memory scores across waves using a published CHARLS cognitive calibration protocol before computing the global score[19].

### Mediators

We tested two parallel mediators. The first mediator was ADL function, computed from six basic (BADL) and five instrumental (IADL) activities of daily living; after harmonized scoring, higher values reflected better function. The second mediator was social and intellectual engagement, built from four social and four intellectual activity items in CHARLS that standardized and summed so that higher scores indicated greater participation. For the primary mediation specification, 2011 data were used. To tighten temporal ordering, a lagged specification repeated the same constructions with 2013 data.

### Contextual modifiers

Two contextual modifiers were entered into the analysis: living environment quality (LEQ) and adverse childhood experiences (ACEs). LEQ was based on five indicators—housing type, indoor temperature adequacy, drinking water source, cooking and heating fuel type, and outdoor PM2.5 exposure dichotomized at the Chinese Ministry of Ecology and Environment threshold of 35.0 μg/m^3^. We summed these scores into a 0-to-6 composite, with higher scores indicating greater cumulative environmental adversity. We then trichotomized the LEQ as favorable (0–1), moderate (2–3), or unfavorable (4–6)[20]. ACEs were gauged with twelve items drawn from the CHARLS 2014 Life History Survey, adapted from established ACE frameworks for the Chinese setting[21]. Each item was coded binary (1 = yes, 0 = no), summed to a score of 0-12, and finally standardized (mean = 0, SD = 1) before being entered into moderation models.

### Covariates

Baseline covariates included age, sex, urban–rural residence, marital status, educational attainment (illiterate, primary school, junior middle school, or senior high school or above), per capita household income (tertiles), smoking status (never, former, or current), drinking frequency (never, moderate, or frequent), physical activity level (low, moderate, or high), sleep duration (≤6 h, 7–8 h, or ≥9 h), and body mass index.

### Statistical analysis

Baseline characteristics were summarized according to 2011 multimorbidity patterns. Continuous variables are presented as means ± standard deviations, and categorical variables as frequencies and percentages. Group differences were assessed using one-way ANOVA, χ^2^ tests, or Fisher’s exact tests, as appropriate. Missing baseline covariates, mediators, ACEs, and LEQ were imputed using an iterative random forest algorithm to generate twenty complete datasets, and estimates were pooled according to Rubin’s rules[22]. Missing repeated cognitive outcomes were handled using full information maximum likelihood in LGCM-SEM and maximum likelihood estimation in LMMs.

We fitted latent class analysis (LCA) models using the poLCA package (version 1.6) for R, with the fifteen binary chronic conditions entered as class indicators. Models with one to six classes were estimated via the expectation-maximization (EM) algorithm with maximum likelihood, using 100 random sets of starting values (maximum iterations = 5,000; convergence tolerance = 1×10^−8^) to reduce the risk of local maxima. Class selection balanced the Bayesian information criterion (BIC), adjusted BIC (aBIC), average posterior probability (AvePP), class size, and clinical interpretability(Supplementary Table 1)[23]. The final five-class solution showed acceptable classification quality, with an entropy of 0.607 and an overall AvePP of 0.751 (class-specific AvePPs ranged from 0.662 to 0.794)(Supplementary Table 2, Panel C). Participants were assigned to the class with the highest posterior probability (modal assignment rule). The five classes were labeled according to their distinctive conditional probability profiles(Fig. S1 and Supplementary Table 2, Panel A) as Minimal morbidity, Cardiometabolic, Extensive multisystem, Sensory-psychiatric-musculoskeletal multimorbidity, and Respiratory-psychiatric-sensory multimorbidity. For brevity, we refer to these hereafter as Minimal morbidity, Cardiometabolic, Extensive multisystem, Sensory-PMM, and Respiratory-PSS(psychiatric–sensory). For time-varying analyses, separate LCAs were fitted at each follow-up wave (2013, 2015, 2018, 2020) with identical specifications; wave-specific classes were labeled by manual comparison of conditional probability profiles against the baseline class structure(Supplementary Table 2, Panels B and C).

LGCMs served to relate baseline multimorbidity profiles to cognitive trajectories between 2011 and 2020[24]. The intercept represented baseline cognitive level, and the slope represented cognitive change over time. Time scores were set as 0, 2, 4, 7, and 9. Three models were fitted: Model 1 adjusted for age and sex; Model 2 further adjusted for urban-rural residence, marital status, education, and household income; and Model 3 additionally included smoking, drinking, physical activity, sleep duration, and BMI. Model fit was assessed using CFI, TLI, RMSEA, and SRMR.

Parallel pathway analyses within the LGCM-SEM framework examined whether ADL function and social and intellectual engagement explained the associations between multimorbidity patterns and cognitive intercept and slope[25, 26]. These analyses were implemented using the lavaan package (version 0.6) with robust maximum likelihood (MLR) estimation and full information maximum likelihood (FIML) for missing cognitive outcomes. Indirect effects were computed using the product-of-coefficients method (a-path × b-path) with standard errors approximated by the multivariate delta method. Two specifications were compared: Model 1 without residual covariances between cognitive indicators, and Model 2 with residual covariances between adjacent-wave indicators; Model 2 was retained as the final specification (Table 3). All mediation estimates were pooled across 20 multiply imputed datasets using Rubin’s rules. Lagged mediation analyses used 2013 mediators and cognitive outcomes from 2015 to 2020, adjusted for baseline cognition, applying a two-stage linear mixed-effects model (LMM) approach via the nlme package: multimorbidity patterns predicted 2013 mediators (a-paths), and 2013 mediators in turn predicted subsequent cognitive trajectories in an LMM with random intercepts and slopes (b-paths). Lagged indirect effects used the product-of-coefficients method with delta-method standard errors, pooled across imputations. Moderation analyses tested the modifying effects of LEQ, ACEs, and their interaction. Supplementary linear mixed-effects models[27] treated wave-specific multimorbidity patterns as time-varying exposures.

Sensitivity analyses were conducted by repeating the primary LGCM using the four-class LCA solution and by performing a complete-case analysis excluding physical activity and household income because of their high missing rates. All analyses were performed using R version 4.4.3[28]. All tests were twosided, with P<0.05 considered statistically significant.

## Results

### Multimorbidity pattern identification and sample characteristics

Supplementary Table 1 shows the fit indices for the one- to six-class LCA models in 2011. Although the four-class solution had the lowest BIC, its advantage over the five-class solution was minimal. The five-class solution had a lower aBIC and additionally identified an Extensive multisystem class that was not separated in the four-class model. This model also showed acceptable classification quality, with an entropy of 0.607 and an average posterior probability of 0.751. Wave-specific LCAs from 2013 to 2020 showed similar classification stability, with average posterior probabilities ranging from 0.724 to 0.751. Therefore, the five-class solution was used in the primary analyses, and the four-class solution was retained for sensitivity analyses.

In 2011, participants were classified into Minimal morbidity (57.0%, n=3,410), Sensory-PMM (20.2%, n=1,208), Cardiometabolic (14.0%, n=837), Extensive multisystem (6.4%, n=381), and Respiratory-PSS (2.4%, n=143). Minimal-morbidity participants were younger, more educated, and had higher baseline cognitive scores. Sensory-PMM participants were more often women, rural residents, and less educated, with lower social and intellectual engagement. Cardiometabolic participants had the highest BMI but baseline cognition comparable to the Minimal morbidity group. Respiratory-PSS participants were the oldest, predominantly male, and had poorer living environments and greater functional limitations.

Extensive multisystem participants had the lowest baseline cognitive scores and greater socioeconomic and functional disadvantages. Baseline characteristics differed significantly across multimorbidity patterns, including age, sex, education, residence, household wealth, BMI, sleep duration, social and intellectual engagement, ADL/IADL function, living environment quality, and baseline cognition. For detailed characteristics, see Table 1 and Fig. S1.

### Association between baseline comorbidity patterns and cognitive trajectories

The unconditional LGCM revealed a linear downward drift in overall cognition from 2011 to 2020, with a mean intercept of 12.87 and an average annual decrease of 0.247 points. Significant intercept and slope variances indicated considerable individual heterogeneity in the starting level and pace of change. The two were moderately positively correlated (r=0.307), suggesting that participants who began higher tended to decline more slowly. A linear specification was retained because the quadratic term showed limited pattern-related variation. The conditional LGCM fit well (Model 3: CFI=0.959, TLI=0.935, RMSEA=0.039, SRMR=0.014).

The groups differed markedly in baseline cognition. In Model 1, Sensory-PMM, Extensive multisystem, and Respiratory-PSS all fell significantly below Minimal morbidity, whereas Cardiometabolic did not. After full adjustment in Model 3, Sensory-PMM still carried the heaviest cognitive penalty (β=−0.757, 95% CI: −0.922 to −0.593), followed by Extensive multisystem (β=−0.469, 95% CI: −0.726 to −0.212); Cardiometabolic and Respiratory-PSS no longer separated from Minimal morbidity. Scaled to the unconditional intercept SD of 2.30, these differences corresponded to Cohen’s d values of −0.33 for Sensory-PMM (small-to-moderate effect) and −0.20 for Extensive multisystem (small effect).

**Figure 2:**
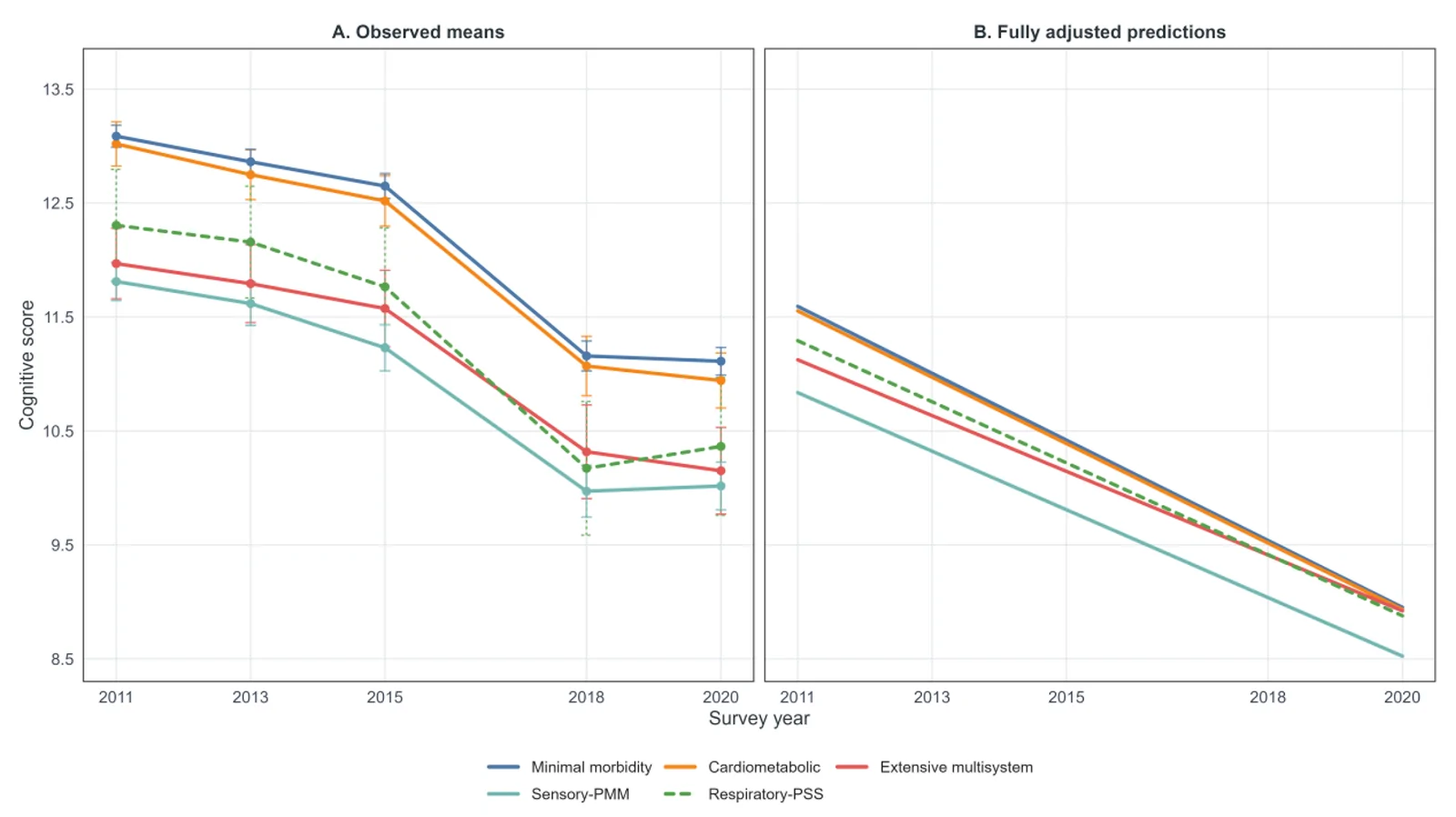
Cognitive trajectories by multimorbidity pattern in CHARLS 2011–2020. Panel A shows unadjusted observed group means (solid points) across five survey waves. Panel B shows latent growth curve model (LGCM) predicted trajectories from the fully adjusted Model 3, with shaded ribbons representing 95% confidence intervals. The dashed line indicates the Respiratory–Psychiatric–Sensory–Multimorbidity group. Time scores were set to 0 (2011), 2 (2013), 4 (2015),7 (2018), and 9 (2020) to reflect unequal survey intervals.

Slopes told a different story. Extensive multisystem declined most slowly (β=0.049, P=0.012), with Sensory-PMM close behind (β=0.037, P=0.001); the slopes for the other two groups were not significant. This low-intercept, gentle-slope configuration suggests limited room for further decline among high-burden groups (Fig. 2). Model 3 explained 44.3% of the intercept variance and 21.2% of the slope variance (Fig. S2). Among the covariates, older age and rural residence predicted both lower baseline cognition and faster decline, whereas higher education predicted better baseline cognition and slower decline.

### Mediating effects of functional and social pathways

**Figure 3:**
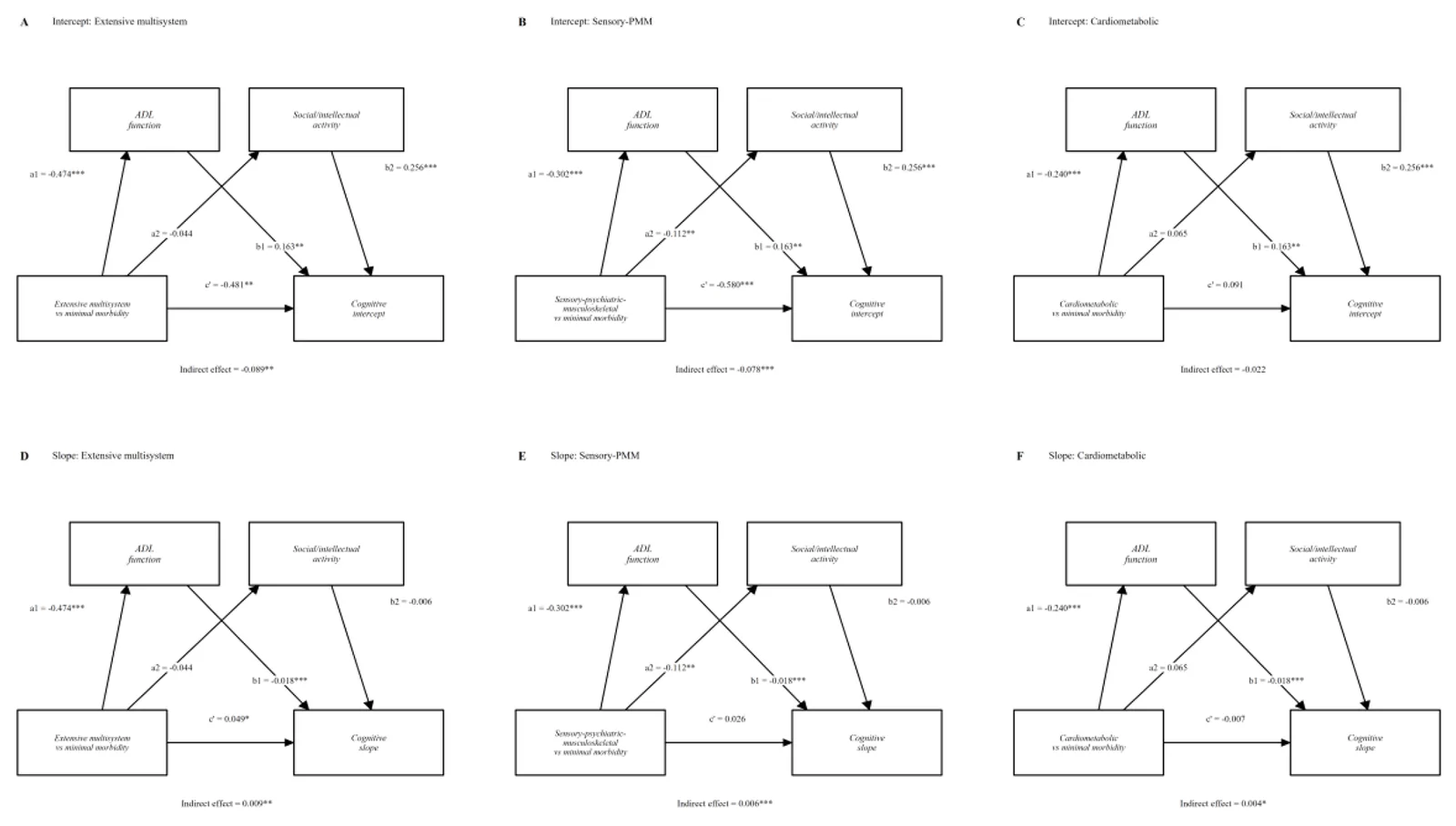
Parallel mediation of baseline multimorbidity patterns on cognitive intercept and slope through ADL function and social/intellectual engagement. Panels A–C depict mediation of the cognitive intercept; panels D–F depict mediation of the cognitive slope. For each panel, the exposure is the multimorbidity pattern versus Minimal morbidity (reference), with ADL function and social/intellectual activity as parallel mediators. Path coefficients are standardized regression weights with significance indicated: *P < 0.05, **P < 0.01, ***P < 0.001. a1 = effect of exposure on ADL function; a2 = effect of exposure on social/intellectual activity; b1 = effect of ADL function on cognition; b2 = effect of social/intellectual activity on cognition; c′ = direct effect of exposure on cognition; total indirect effect = a1×b1 + a2×b2. PMM = psychiatric–musculoskeletal. PSS = psychiatric–sensory.

We next asked whether ADL function and social and intellectual engagement jointly mediate the effects of multimorbidity patterns on cognitive trajectories (Fig. 3).

#### Baseline mediation

At baseline, all nonreference patterns were associated with poorer ADL function, which in turn predicted higher cognitive intercepts. Indirect pathways through ADL function were significant across all four patterns. Social and intellectual engagement showed a more selective pattern: only Sensory-PMM was associated with reduced engagement, generating a significant indirect association with baseline cognition. Total indirect effects on the cognitive intercept were significant for Extensive multisystem (indirect effect=−0.089, 95% CI: −0.146 to −0.031; P=0.004) and Sensory-PMM (indirect effect=−0.078, 95% CI: −0.114 to −0.042; P<0.001). Their direct effects remained significant, indicating partial rather than full mediation. For the cognitive slope, total indirect effects were significant for Cardiometabolic, Extensive multisystem, and Sensory-PMM, but not for Respiratory-PSS.

#### Lagged mediation

In the lagged analysis, 2013 ADL function and social and intellectual engagement both predicted higher cognitive levels from 2015 to 2020, but neither predicted the rate of cognitive change. Indirect effects on subsequent cognitive levels remained significant for Extensive multisystem (indirect effect=−0.081, 95% CI: −0.134 to −0.029; P=0.004) and Sensory-PMM (indirect effect=−0.070, 95% CI: −0.099 to −0.041; P<0.001), whereas Respiratory-PSS showed only marginal evidence of mediation. Cardiometabolic showed opposite-direction pathways, with poorer ADL function but higher engagement, which canceled each other out and yielded a nonsignificant total indirect effect. Even after accounting for both mediators, Sensory-PMM still showed a lower cognitive intercept and slower decline than Minimal morbidity, suggesting that other unmeasured pathways may also be involved.

#### Moderation by LEQ and ACEs

We tested whether LEQ and ACEs modified the associations between multimorbidity patterns and cognitive trajectories using three LGCM-SEM specifications: an LEQ-only model, an ACE-only model, and a combined LEQ+ACE model. In the combined model, most VIFs were modest, although higher values were observed for Extensive multisystem (VIF=7.29), Sensory-PMM (VIF=6.72), and ACEs (VIF=5.39). No VIF exceeded 10, indicating no severe multicollinearity.

Compared with favorable LEQ, unfavorable LEQ was associated with a lower cognitive intercept (β=−0.532, 95% CI: −0.849 to −.214; P=0.002), whereas moderate LEQ was not. Neither moderate nor unfavorable LEQ was associated with the cognitive slope. ACEs were not significantly associated with either the intercept or slope.

**Figure 4 :**
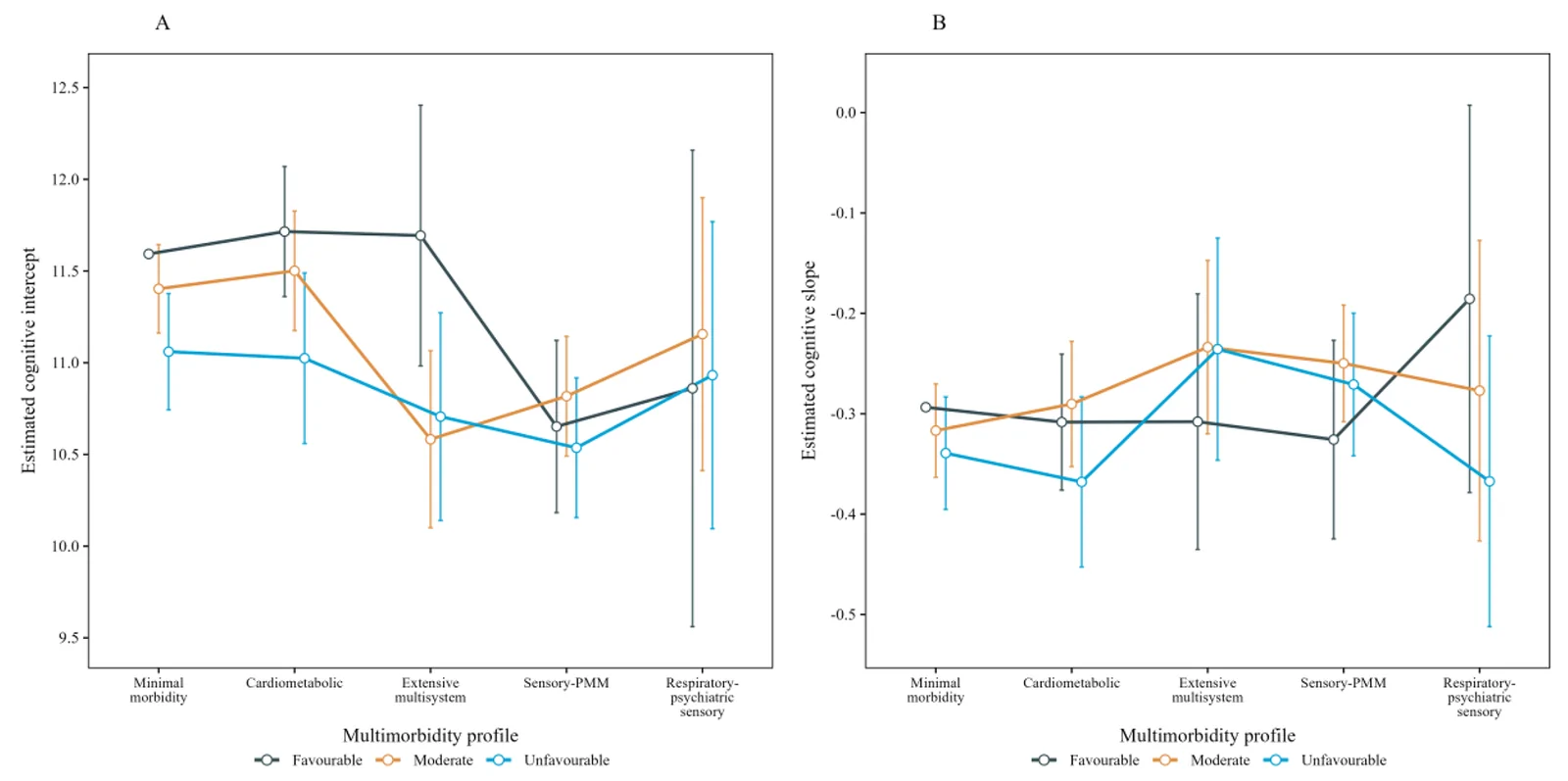
Moderating effect of living environment quality (LEQ) on the association between multimorbidity patterns and cognitive trajectories Panel A shows estimated cognitive intercepts; Panel B shows estimated cognitive slopes. Points represent marginal estimated means from the fully adjusted latent growth curve model–structural equation model (LGCM-SEM), with error bars indicating 95% confidence intervals. LEQ was trichotomized as favourable (0–1), moderate (2–3), or unfavourable (4–6). The significant interaction between moderate LEQ and Extensive multisystem on the cognitive intercept (β = −0.900, P = 0.040) is highlighted. PMM = psychiatric–musculoskeletal. PSS = psychiatric–sensory.

For interaction terms, only the interaction between moderate LEQ and Extensive multisystem was significantly associated with the cognitive intercept (β=−0.900, 95% CI: −1.756 to −0.044; P=0.040). LEQ interactions with Sensory-PMM on the slope showed marginal trends, whereas all ACE × pattern interactions and LEQ × ACE interactions were nonsignificant. Overall, evidence for moderation was limited (Fig. 4).

### Supplementary: Time-varying multimorbidity and cognitive trajectories

We further used linear mixed-effects models to examine whether wave-specific multimorbidity patterns were associated with cognitive change during follow-up.

In the concurrent model, Extensive multisystem participants had lower cognitive scores than Minimal morbidity participants at a given wave (β=−0.395, 95% CI: −0.611 to −0.179; P<0.001), as did those in the Sensory-PMM group (β=−0.277, 95% CI: −0.395 to −0.158; P<0.001). Cardiometabolic showed only a borderline association with higher concurrent cognitive scores (β=0.140, 95% CI: 0.000 to 0.279; P=0.050), whereas Respiratory-PSS was not significantly associated with cognition.

For pattern × time interactions, only Cardiometabolic was associated with faster cognitive decline over time (β=−0.028, 95% CI: −0.052 to −0.005; P=0.019). No other pattern × time interaction was significant. In three-way models, Sensory-PMM and Extensive multisystem remained associated with lower cognitive scores, whereas all pattern × time × urbanicity interactions were nonsignificant, providing little evidence of urban-rural heterogeneity. In the lagged model adjusted for prior-wave cognition, only Sensory-PMM was associated with lower cognitive performance in the next wave (β=−0.174, 95% CI: −0.268 to −0.079; P<0.001). Full results are shown in Supplementary Table 5 and Figs. S3–S4.

### Sensitivity analyses

The four-class sensitivity analysis produced results broadly consistent with the primary LGCM. In Model 3, Sensory-PMM remained associated with the largest baseline cognitive disadvantage (β=−0.540, 95% CI: −0.70 to −0.38; P<0.001) and a slower decline (β=0.036, P<0.001). Cardiometabolic and Respiratory-PSS were not significantly different from Minimal morbidity. Because the four-class solution did not separate Extensive multisystem as an independent class, its class-specific association could not be directly assessed.

Complete-case analyses yielded similar findings. After excluding physical activity and household income because of high missingness, 4,991 participants were retained. Sensory-PMM again showed the strongest baseline disadvantage (β=−0.845, 95% CI: −1.02 to −0.67; P<0.001) and a slower decline (β=0.045, P<0.001). Extensive multisystem also retained a lower intercept (β=−0.607, P<0.001) and a gentler slope (β=0.060, P=0.004), whereas Cardiometabolic and Respiratory-PSS remained nonsignificant. Full results are shown in Supplementary Table 6.

## Discussion

In this nine-year longitudinal study of 5,979 community-dwelling Chinese older adults, we identified five baseline multimorbidity patterns and examined their associations with cognitive trajectories. Several findings stood out. Sensory-PMM and Extensive multisystem had the lowest cognitive baselines, whereas Cardiometabolic and Respiratory-PSS did not differ from Minimal morbidity after full adjustment. Decline rates were more nuanced: the two high-burden groups showed attenuated slopes, consistent with floor effects, while Cardiometabolic participants showed faster decline in time-varying models. ADL function and social/intellectual engagement partially explained these links, and living environment quality showed limited but meaningful contextual modification, particularly for Extensive multisystem.

Our findings support the growing view that multimorbidity research should move beyond simple disease counts toward clinically meaningful phenotypes[4, 29]. Sensory-PMM, encompassing sensory impairment, psychiatric conditions, and musculoskeletal disorders, had the strongest and most consistent association with low baseline cognition. This pattern is biologically and socially plausible: sensory loss narrows incoming information, psychiatric symptoms sap motivation and social participation, and chronic pain weakens physical function and nutritional status[30, 31]. Together, these processes may erode cognitive reserve and reduce stimulating experiences. Extensive multisystem disease, spanning several organ systems, carried a similarly heavy cognitive burden, most likely through cumulative physiological dysregulation and depletion of reserves[32]. By contrast, Cardiometabolic conditions did not show a consistent baseline cognitive disadvantage in the primary LGCM, suggesting that cognitive risk depends not only on disease burden but also on how diseases cluster and shape functional and psychological well-being.

The low-intercept, gentle-slope signature observed in Sensory-PMM and Extensive multisystem should be interpreted as being consistent with a floor effect rather than a protective mechanism[33]. When baseline cognition is already low, there is simply less territory left to lose, so slope differences may flatten out. This finding is consistent with cumulative disadvantage theory, which argues that health inequalities crystallize early and then cast long shadows across the life course[34]. The corresponding Cohen’s d values were small to moderate, indicating that these baseline differences were clinically meaningful rather than merely statistically significant[35]. Even modest cognitive deficits can disrupt chronic disease self-management, medication adherence, and doctor–patient communication[36]; therefore, identifying individuals with low cognitive baselines may be as important as tracking rapid decliners.

Dynamic analyses added another layer. Concurrent models reaffirmed that Sensory-PMM and Extensive multisystem were associated with lower cognitive levels. Cardiometabolic participants, however, showed no baseline disadvantage in the primary LGCM but displayed faster decline in time-varying models. This pattern suggests that vascular and metabolic insults may leave cognition relatively intact initially, only to erode it progressively, which is consistent with the vascular contributions to cognitive impairment and dementia hypothesis[37]. These temporal patterns were similar in urban and rural participants. In lagged models, only Sensory-PMM remained associated with lower cognition at the next wave, suggesting that this pattern may be a particularly stable harbinger of cognitive difficulty, possibly because its sensory and psychiatric components create persistent barriers to cognitive stimulation and social engagement[38].

The pathway analyses further clarified these associations. ADL function appeared to be a common pathway across all nonreference patterns, suggesting that functional capacity may be central to the multimorbidity–cognition link. Social and intellectual engagement was more selective: it mediated the association mainly for Sensory-PMM. This finding suggests that reduced engagement is not merely a consequence of multimorbidity in general, but may be especially tied to patterns involving sensory and psychiatric burden.An especially notable finding was observed in the Cardiometabolic group: ADL function decreased, whereas engagement improved, and the two countervailing pathways neutralized each other. One interpretation is that some individuals facing cardiovascular and metabolic conditions may increase cognitive and social engagement as a compensatory form of behavioral self-management, consistent with self-efficacy theory[39] and the patient health engagement model[40]. This compensatory capacity, however, may not be equally available to all groups. In Sensory-PMM, sensory impairment and psychiatric symptoms may simultaneously erode function and limit the ability to mobilize compensatory behaviors.

Living environment quality also mattered. Unfavorable LEQ was associated with substantially lower baseline cognition, in line with evidence linking substandard housing, air pollution, and infrastructure deficits to cognitive harm through inflammation, restricted mobility, and reduced social participation [41, 42]. LEQ was not associated with the rate of decline, suggesting that environmental conditions may shape the cognitive starting point more than later deterioration. The interaction between moderate LEQ and Extensive multisystem was significant, indicating that even moderately deprived environments may amplify the cognitive burden of extensive multisystem disease. This is compatible with the environmental vulnerability hypothesis[43]. ACEs, in contrast, showed neither main nor moderating effects, and no LEQ × ACE interaction was observed. Taken together, these findings suggest that current environmental conditions may play a more detectable role than distal early-life adversity in this cohort[44].

These findings have several clinical and policy implications. First, cognitive risk stratification in multimorbidity should move beyond crude disease counts toward pattern-sensitive screening. Older adults in the Sensory-PMM and Extensive multisystem groups may warrant earlier cognitive and functional assessment. Second, interventions should be pattern-calibrated. Maintaining ADL function is a universal goal, but the route may differ: Sensory-PMM may benefit most from integrated management of hearing and vision loss, depression, pain, and social isolation, whereas Cardiometabolic participants may benefit from structured cognitive and social programs that build on compensatory engagement.Third, cognitive health strategies should not stop at the clinic door. Housing upgrades, clean fuel access, and air-quality improvement may need to be incorporated into chronic disease care, particularly for older adults with extensive multisystem burden. Daily living skills training and physical rehabilitation may help preserve function. Psychological counseling and social support may also be incorporated into chronic disease care[14].

Sensitivity checks supported the robustness of the findings. Collapsing the LCA to four classes left the strong baseline association of Sensory-PMM and slower decline intact, although the independent signal of Extensive multisystem could not be assessed because this class was not separated in the four-class solution. Similarly, the complete-case analysis, which omitted physical activity and household income because of high missingness and retained 4,991 participants, produced materially similar results. These findings suggest that neither the class-number choice nor the missing-data strategy drove the main conclusions.

Several limitations should be noted. Chronic conditions were ascertained mainly through self-reported physician diagnosis and medication use, which may introduce recall bias and misclassification. The observational design also limits causal inference. In the primary pathway analysis, exposure, mediators, and baseline cognition were measured in the same wave; although lagged models strengthened temporal ordering, reverse causation and residual confounding cannot be ruled out. Therefore, the results should be interpreted as longitudinal associations rather than causal effects. In addition, LCA is data driven, and class structure may vary according to the selected conditions, sample composition, and class number. Although posterior probabilities were acceptable, some classification uncertainty remains inherent to LCA. The small Respiratory-PSS group particularly requires external validation. Finally, mortality may compete with cognitive decline, as frailer participants may die before further cognitive deterioration is observed, raising the possibility of selection bias.

## Supplementary Material

This is a list of supplementary files associated with this preprint. Click to download.

- SFigureTables.docx
- Tables.docx

Tables 1 to 4 are available in the supplementary files section.

## Data Availability

The datasets used and analyzed during the current study are available from the corresponding author upon reasonable request.
